# Gender differences in the association between weight-adjusted waist index and migraine: A cross-sectional study

**DOI:** 10.1371/journal.pone.0323087

**Published:** 2025-05-16

**Authors:** Shulong Liu, Jiangting Li, Guobo Xie

**Affiliations:** 1 Jiangxi Provincial Chest Hospital, Nanchang, Jiangxi, China; 2 Jiangxi Maternal and Child Health Hospital, Nanchang, Jiangxi, China; 3 Department of Cardiology, Jiangxi Provincial People’s Hospital Affiliated to Nanchang University, Nanchang, Jiangxi, China; University of Diyala College of Medicine, UNITED STATES OF AMERICA

## Abstract

**Objective:**

This study examines how weight-adjusted waist index (WWI) correlates with the occurrence of migraine in U.S. adults.

**Background:**

Being overweight significantly increases the likelihood of experiencing migraines; nonetheless, conventional metrics like waist circumference (WC) and body mass index (BMI) might not completely capture the level of migraine risk tied to obesity. WWI integrates the strengths of WC while minimizing its correlation with BMI, which might make it a more accurate indicator of central obesity-related migraine susceptibility.

**Methods:**

This study performed a cross-sectional analysis using data from 9,688 participants obtained from the National Health and Nutrition Examination Survey (NHANES), covering the years 1999–2004. Migraine occurrence was evaluated through questionnaires, and participants’ WWI was computed. Weighted multivariable logistic regression models were used to examine the association between WWI and migraines. Restricted cubic splines (RCS) were applied to evaluate the dose-response relationship between WWI and migraines. Furthermore, interaction tests and subgroup analyses were executed. The receiver operating characteristic (ROC) curve, paired with DeLong et al.’s test, was employed to compare the predictive power of WWI, BMI, and WC for migraines.

**Results:**

The overall prevalence of migraines was found to be 21.50% (weighted population: 31,888,075 out of 148,278,824). In Model 3, the link between WWI and migraines in women showed no statistical significance (OR = 0.94, 95% CI: 0.82–1.07). In this model, each unit increase in WWI among men was linked to a 22% higher risk of migraines (OR = 1.22, 95% CI: 1.05–1.42). When stratified by quintiles, individuals in the third quintile (Q3) displayed a 69% higher likelihood of experiencing migraines compared to those in the first quintile (Q1) (OR = 1.69, 95% CI: 1.19–2.40), with a significant inflection point observed at 10.95 cm/√kg. Significant interactions were noted among various age groups (p for interaction = 0.018). WWI demonstrated a stronger predictive capability for migraine compared to BMI and WC.

**Conclusion:**

A U-shaped positive correlation of WWI with migraines was observerd among adult males in the U.S., while no significant correlation was found in females. Within the context of BMI and WC, WWI exhibited a superior predictive capacity for migraines.

## 1. Introduction

Migraine is a common neurological disorder involving recurrent throbbing headaches, often accompanied by nausea, vomiting, and sensitivity to light and sound [1]. The World Health Organization (WHO) ranks migraine as the second most disabling neurological disorder and the third most prevalent disease globally [2,3].Global Burden of Disease Study results show that migraines negatively affect the quality of life for over one billion people worldwide [4]. In the United States, migraine affects an estimated 18.2% of the female population and 6.5% of males, with 23% of households demonstrating at least one affected individual [5]. Although the prevalence of migraine varies across different age and gender groups, it remains a pressing global health issue. Despite extensive research efforts by the medical community, the pathophysiological mechanisms that contribute to migraine remain not entirely understood. Consequently, a comprehensive understanding of migraine and its associated factors has become increasingly urgent for the effective prevention, management, and enhancement of the prognosis and quality of life for migraine patients [6].

Obesity is a key risk factor for migraine development [7]. Commonly used indices related to obesity include BMI and WC. However, recent research indicates that people classified as overweight or obese may have all-cause mortality rates that are comparable to, or even lower than, those of individuals with a normal weight [8–10]. The health impact of overweight and obesity remain debated, partly because these anthropometric measurements fail to distinctly separate muscle mass from fat mass [11,12]. Studies on diabetes and cardiovascular diseases suggest central obesity is a better predictor of disease risk and mortality than BMI-based general obesity [13–15]. WWI, an indicator of obesity that has gained attention in recent year, was first proposed by Park and colleagues at Korea University College of Medicine in Seoul. WWI adjusts WC for weight, combining WC’s benefits while reducing its confounding link with BMI [16]. This index not only differentiates between muscle mass and fat distribution but also addresses the issue of central obesity independently of body weight [17,18]. Studies have shown that elevated WWI is strongly linked to diseases such as stroke, osteoporosis, cognitive impairment, and depression [19–22]. Although WWI has proven effective in predicting the risk of various diseases, the potential connection between WWI and migraines remains unexplored in existing research. Consequently, we carried out a cross-sectional analysis utilizing information from NHANES conducted from 1999 to 2004 to examine the potential impact of WWI on migraine prevalence among U.S. adults.

## 2. Methods

### 2.1. Study Design

NHANES represents an ongoing cross-sectional research initiative carried out by CDC within the United States [23]. NHANES employs a stratified, multistage probability sampling method to ensure a highly representative sample of U.S. children and adults for assessing health and nutrition status [24]. The survey operates on a recurring two-year cycle and includes a diverse range of data, such as demographic details, dietary evaluations, physical assessments, laboratory analysis, and various other health questionnaires. The study protocol was approved by the National Center for Health Statistics (NCHS) Research Ethics Review Board, and all participants provided written informed consent [25].The NHANES Institutional Review Board (prior to 2003)/NCHS Research Ethics Review Board (2003 and after) approved the NHANES 1999–2004 (NHANES 1999–2004 Protocol #98–12)(https://www.cdc.gov/nchs/nhanes/about/erb.html).

### 2.2. Study population

**Fig 1:**
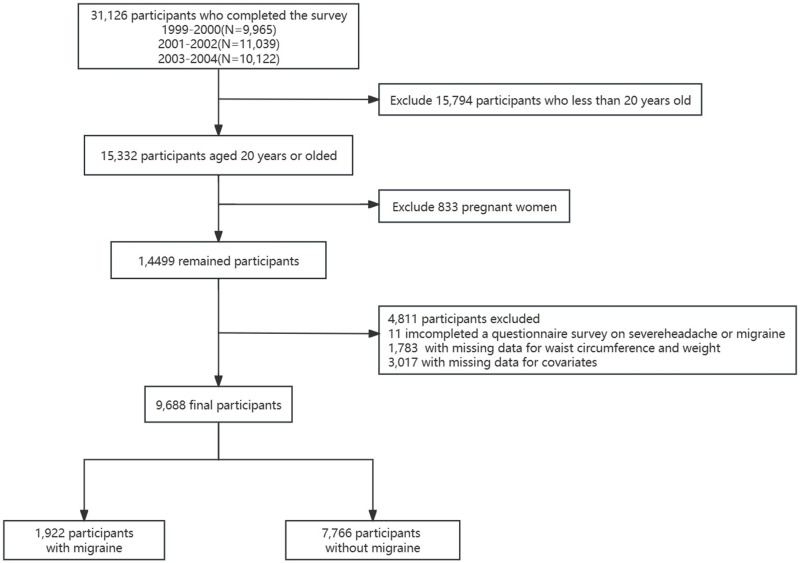
Inclusion and exclusion flow chart.

Information on migraines and severe headaches was collected exclusively during the NHANES cycles from 1999 to 2004. Consequently, our cross-sectional study is constrained to data from this six-year period. A total of 31,126 participants completed the interviews, of which 15,794 were under the age of 20. From the remaining 15,332 participants, we further excluded pregnant women (n = 833), individuals with missing migraine data (n = 11), those lacking WC and weight data (n = 1,783), and those with incomplete covariate information (n = 3,017). Thus, the final sample comprised 9,688 participants (Fig 1). Data from this study is publicly accessible at https://www.cdc.gov/nchs/nhanes/.

### 2.3. Assessment of WWI

WWI is a novel measure of central obesity, with higher values indicating a greater central obesity. This index is derived by measuring WC (cm) and dividing it by the square root of body mass (kg) (WWI = WC/√weight) [26].Certified health professionals conducted anthropometric assessments at mobile examination sites, ensuring precise data collection by trained staff. Weight measurements for participants were obtained using a digital scale while they stood barefoot and wore light clothing. WC was measured at the intersection of the midaxillary line and the horizontal plane above the right iliac crest using a tape measure [27]. In this research, WWI was considered as an exposure variable and analyzed both continuously and by quintiles.

### 2.4. Assessment of migraine

The evaluation of migraines was performed utilizing the NHANES Pain Questionnaire (MPQ). In question 090 of the MPQ, participants were queried, “In the past three months, have you experienced severe headaches or migraines?” Those who answered “Yes” were classified as individuals with migraines. Findings from the American Migraine Prevalence and Prevention (AMPP) study [28], of the participants, 17.4% self-reported “severe headaches,” 11.8% of the patients met the criteria of the International Classification of Headache Disorders, 2nd Edition (ICHD-II), and 4.6% of the patients met the criteria for “probable migraine.” Therefore, it is reasonable to classify patients reporting severe headaches or migraines as suffering from migraines. This diagnostic criterion is widely used in current epidemiological studies [29,30].

### 2.5. Covariates of interest

Based on existing literature and clinical experience, we identified potential confounders to include as covariates that may affect the relationship between WWI and migraines. These covariates encompass gender, age (categorized as less than 60 years and 60 years or older), race/ethnicity (Mexican American, non-Hispanic white, non-Hispanic black, other Hispanic,other races), educational level (less than high school, high school, above high school), marital status (married, living alone, living with a partner), poverty income ratio (PIR) (≤1.30, 1.31–3.50, > 3.50), smoking status (never smoked, former smoker, or current smoker), alcohol consumption (yes or no), hemoglobin count, C-reactive protein count, total cholesterol level, and the existence of diabetes, hypertension, kidney failure, and cardiovascular diseases. For the purposes of this study, cardiovascular disease is defined as experiencing one or more of the following conditions: coronary heart disease, heart attack, congestive heart failure, stroke, or angina.

### 2.6. Statistical analysis

Our study meticulously accounted for the intricate sampling framework and the corresponding sample weights for different study periods, as detailed in the NHANES analytic guidelines [31]. Consequently, our statistical estimates are more representative. For the integrated analysis of the NHANES data from 1999–2000 and 2001–2002, we utilized the four-year MEC weight (WTMEC 4YR), while for the 2003–2004 data, we applied the two-year MEC weight (WTMEC 2YR). Following NHANES guidelines, we calculated sampling weights for 1999–2004 by applying 2/3 to the 1999–2002 weight and 1/3 to the 2003–2004 weight.

Continuous variables were expressed as weighted means (standard deviation) or medians (interquartile range), while categorical variables were summarized as frequencies and percentages. T-tests were used for continuous variables and χ² tests for categorical variables to compare baseline characteristics of individuals with and without migraines. The relationship between WWI and migraines was examined with weighted multivariable logistic regression across 3 distinct models to calculate odds ratios (OR) and 95% confidence intervals (CI). Model 1 included no covariates. Model 2 adjusted for gender, education, marital status, age, PIR, smoking, race, and alcohol consumption. Model 3 further adjusted for total cholesterol (TC), C-reactive protein (CRP), diabetes, hypertension, kidney failure, hemoglobin (HB), and cardiovascular disease. WWI was categorized into quintiles (Q1: < 10.21; Q2: 10.21–10.71; Q3: 10.72–11.13; Q4: 11.14–11.65; Q5: ≥ 11.66), with Q1 serving as the reference group. Trend tests were used to assess the linear relationship between WWI and migraines. After Model 3 adjustments, weighted restricted cubic splines (RCS) examined linearity and the dose-response relationship. A smoothed piecewise logistic regression approach was employed to investigate the threshold effect of WWI on migraines. Additionally, weighted subgroup analyses were performed to investigate the WWI-migraine relationship across various strata, including age, race, education level, smoking status, alcohol consumption, marital status, diabetes, hypertension, gender, and cardiovascular disease. Interaction tests evaluated the stability of this relationship across different populations. The receiver operating characteristic (ROC) curve and DeLong et al.‘s test compared WWI’s predictive ability for migraines with that of BMI and WC. All statistical analyses used the R software version 4.3.3, with a significance level set at a two-sided p-value <0.05.

## 3. Results

### 3.1. Weighted baseline characteristics

This study included 9,688 individuals from the 1999–2004 NHANES dataset. When the complex sampling design of NHANES was taken into consideration, the weighted population for the study totaled 148,278,825 people, with an average age of 46.03 years (±16.38) Among these participants, 31,888,075 individuals (21.50%) were identified as migraine sufferers, with a male proportion of 49.66%. The mean Weight-Adjusted-Waist Index (WWI) for all participants was 10.79 ± 0.81 cm/√kg. In comparison to individuals without migraine, those suffering from migraines were younger and exhibited a significantly higher prevalence of females. Furthermore, they had a lower proportion of non-Hispanic whites and married individuals, as well as lower educational attainment and income levels. Additionally, migraine sufferers were more likely to smoke and consume alcohol. They also demonstrated a higher prevalence of renal failure, lower levels of hemoglobin and total cholesterol, and elevated levels of CRP. However, there were no significant differences noted in the mean WWI or in the prevalence of diabetes, hypertension, and cardiovascular diseases (Table 1).

**Table 1:** Weighted baseline characteristics of all participants by migraine.

Characteristic	Overall	Without migraine	Migraine	p
n	148278824.51	116390749.4	31888075.11	
WWI, Mean ± SD	10.786 (0.806)	10.792 (0.816)	10.767 (0.767)	0.335
Sex, n (%)				
Male	73635078.81 (49.66)	62270130.48 (53.50)	11364948.33 (35.64)	<0.001
Female	74643745.70 (50.34)	54120618.92 (46.50)	20523126.78 (64.36)	
Age, Mean ± SD	46.030 (16.382)	47.178 (16.876)	41.840 (13.645)	<0.001
Race, n (%)				
Mexican American	10050827.42 (6.78)	7732471.96 (6.64)	2318355.47 (7.27)	0.030
Other Hispanic	8004732.88 (5.40)	5795255.60 (4.98)	2209477.28 (6.93)	
Non-Hispanic white	110177556.13 (74.30)	87620449.98 (75.28)	22557106.15 (70.74)	
Non-Hispanic black	14115087.69 (9.52)	10638270.84 (9.14)	3476816.85 (10.90)	
Others	5930620.38 (4.00)	4604301.02 (3.96)	1326319.36 (4.16)	
Education level, n (%)				
Less than high school	27214868.86 (18.35)	20178317.15 (17.34)	7036551.72 (22.07)	<0.001
High school	38571850.46 (26.01)	29598804.59 (25.43)	8973045.86 (28.14)	
More than high school	82492105.18 (55.63)	66613627.66 (57.23)	15878477.52 (49.79)	
Marital status, n (%)				
Married	88977658.22 (60.01)	71173146.11 (61.15)	17804512.11 (55.83)	<0.001
Living alone	50688837.03 (34.18)	39505842.54 (33.94)	11182994.49 (35.07)	
Living with a partner	8612329.25 (5.81)	5711760.75 (4.91)	2900568.50 (9.10)	
PIR, Mean ± SD	3.055 (1.611)	3.164 (1.598)	2.657 (1.598)	<0.001
Smoke status, n (%)				
Never	73631972.12 (49.66)	57952087.28 (49.79)	15679884.85 (49.17)	<0.001
Former	37533337.57 (25.31)	31196332.07 (26.80)	6337005.50 (19.87)	
Current	37113514.81 (25.03)	27242330.05 (23.41)	9871184.76 (30.96)	
Alcohol status, n (%)				
YES	108654516.69 (73.28)	86826044.77 (74.60)	21828471.92 (68.45)	0.001
NO	39624307.82 (26.72)	29564704.63 (25.40)	10059603.19 (31.55)	
Haemoglobin, Mean ± SD	14.557 (1.434)	14.620 (1.420)	14.324 (1.459)	<0.001
C reactive protein, Mean ± SD	0.409 (0.774)	0.394 (0.791)	0.463 (0.709)	0.001
Total cholesterol, Mean ± SD	5.235 (1.100)	5.248 (1.109)	5.188 (1.064)	0.045
Diabetes, n (%)				
NO	138491998.87 (93.40)	108622825.83 (93.33)	29869173.04 (93.67)	0.517
YES	9786825.64 (6.60)	7767923.57 (6.67)	2018902.07 (6.33)	
Hypertensive, n (%)				
NO	108111244.14 (72.91)	85180316.37 (73.18)	22930927.77 (71.91)	0.239
YES	40167580.37 (27.09)	31210433.03 (26.82)	8957147.34 (28.09)	
Kidney weakness failure, n (%)				
NO	145415714.98 (98.07)	114538794.94 (98.41)	30876920.04 (96.83)	<0.001
YES	2863109.52 (1.93)	1851954.46 (1.59)	1011155.06 (3.17)	
CVD, n (%)				
NO	136107748.96 (91.79)	106869229.27 (91.82)	29238519.69 (91.69)	0.898
YES	12171075.55 (8.21)	9521520.13 (8.18)	2649555.41 (8.31)	

### 3.2. Association between WWI and migraine

Weighted multivariable logistic regression showed no significant association between WWI and migraines across all three models (Table 2). Specifically, the outcomes were as follows: Model 1: OR=0.96, 95% CI: 0.89–1.04; Model 2: OR=1.09, 95% CI: 0.99–1.20; Model 3: OR=1.03, 95% CI: 0.93–1.14. When stratifying the study population by gender, a substantial difference emerged in the relationship between WWI and migraine. For women, Model 1: OR=0.89, 95% CI: 0.81–0.98; Model 2: OR=1.01, 95% CI: 0.89–1.14; Model 3: OR=0.94, 95% CI: 0.82–1.07. In the fully adjusted Model 3, each unit increase in WWI was linked to a 22% higher migraine risk in men (OR = 1.22, 95% CI: 1.05–1.42), indicating a positive correlation. Upon converting WWI into quintiles, participants in the Q2, Q4, and Q5 quintiles exhibited a 15% (OR=1.15, 95% CI: 0.78–1.68), 29% (OR=1.29, 95% CI: 0.87–1.91), and 30% (OR=1.30, 95% CI: 0.82–2.05) increased risk of migraine, respectively, compared to those in Q1; however, these results were not statistically significant (p > 0.05). Participants in Q3 showed a significant 69% higher migraine risk (OR = 1.69, 95% CI: 1.19–2.40, p < 0.05). All trend tests were non-significant (p for trend > 0.05).

**Table 2:** The association between WWI and migraine.

Exposure	Model 1[OR (95% CI)]	Model 2[OR(95% CI)]	Model 3[OR (95% CI)]
All			
WWI(continuous)	0.96(0.89,1.04)	1.09(0.99,1.20)	1.03(0.93,1.14)
WWI(quartile)			
Q1(＜10.21)	1(Ref)	1(Ref)	1(Ref)
Q2(10.21 ~ 10.71)	0.99(0.80,1.22)	1.11(0.88,1.41)	1.08(0.85,1.37)
Q3(10.72 ~ 11.13)	0.97(0.78, 1.19)	1.24(0.98,1.57)	1.17(0.92,1.48)
Q4(11.14 ~ 11.65)	0.94(0.78,1.13)	1.21(0.97,1.51)	1.11(0.88,1.40)
Q5(≥11.66)	0.86(0.67,1.10)	1.1(0.82,1.48)	0.94(0.70,1.25)
p for tend	0.19	0.23	0.954
Female			
WWI(continuous)	0.89(0.81,0.98)	1.01(0.89,1.14)	0.94(0.82,1.07)
WWI(quartile)			
Q1(＜10.21)	1(Ref)	1(Ref)	1(Ref)
Q2(10.21 ~ -10.71)	1.06(0.77,1.45)	1.1(0.78,1.55)	1.06(0.75,1.50)
Q3(10.72 ~ 11.13)	0.89(0.67,1.19)	0.99(0.72,1.35)	0.9(0.66,1.25)
Q4(11.14 ~ 11.65)	0.94(0.75,1.20)	1.11(0.85,1.44)	1(0.76,1.31)
Q5(≥11.66)	0.71(0.53,0.97)	0.95(0.65,1.39)	0.79(0.55,1.14)
p for tend	0.012	0.866	0.192
Male			
WWI(continuous)	1.01 (0.89,1.15)	1.28 (1.11,1.48)	1.22 (1.05,1.42)
WWI(quartile)			
Q1(＜10.21)	1(Ref)	1(Ref)	1(Ref)
Q2(10.21 ~ 10.71)	0.97(0.70, 1.36)	1.16(0.80,1.69)	1.15(0.78,1.68)
Q3(10.72 ~ 11.13)	1.16(0.87, 1.54)	1.7(1.21,2.39)	1.69(1.19,2.40)
Q4(11.14 ~ 11.65)	0.93(0.68, 1.26)	1.37(0.96,1.97)	1.29(0.87,1.91)
Q5(≥11.66)	0.96(0.66, 1.40)	1.49(0.95,2.33)	1.3(0.82,2.05)
p for tend	0.87	0.019	0.085

Model 1: no adjustment for any covariates

Model 2: adjusted for age,race,education level,marital status,PIR,smoking status,alcohol consumption

Model 3: adjusted for Model 2 covariates and HB,CRP,TC, Diabetes, hypertension, kidney failure, CVD

Abbreviations: PIR,ratio of family income to poverty, HB,haemoglobin,CRP, C reactive protein,TC, Total cholesterol, CVD, Cardiovascular disease.

### 3.3. Dose-relationship between WWI and migraine

**Fig 2:**
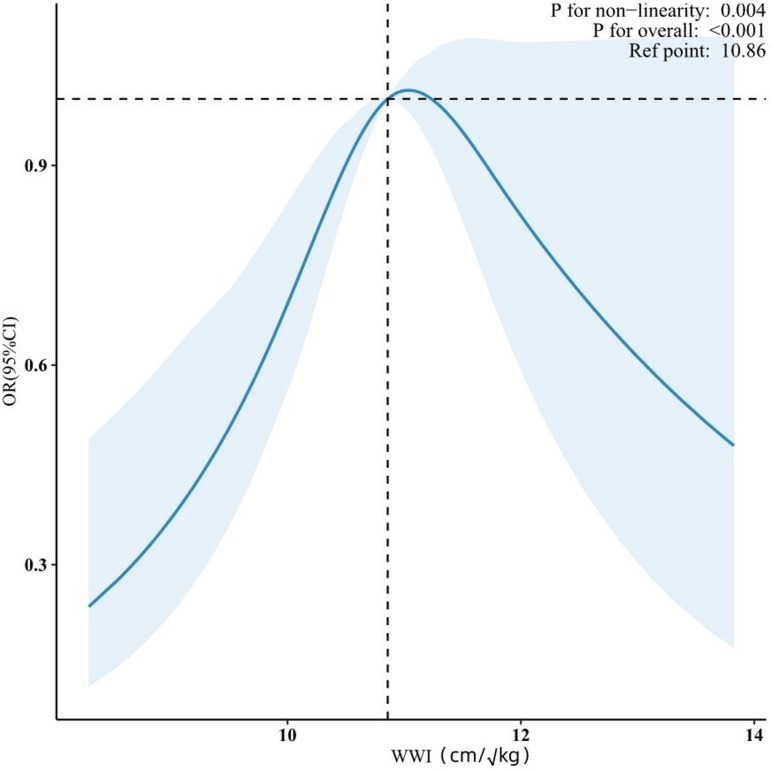
The RCS curve of the association between WWI and migraine among male participants.

In the completely adjusted Model 3, RCS analysis indicated a non-linear, inverted U-shaped dose-response correlation between WWI and the prevalence of migraines in male participants (p for non-linearity = 0.004) (Fig 2). Additionally, a threshold point was identified at 10.95 (95% CI: 10.917–10.983). Prior to this threshold, WWI demonstrated a positive correlation with migraine prevalence (p = 0.015), indicating that each unit increase in WWI was associated with a 36.5% higher risk of migraine (OR=1.365, 95% CI: 1.063–1.753). Beyond this threshold, a negative association was noted (OR=0.927, 95% CI: 0.65–1.32); nevertheless, this connection was not statistically significant (Table 3).

**Table 3:** Threshold effect analysis of the relationship of WWI with migraine among males.

WWI	Adjusted Model	p-value
＜10.95	1.365 (1.063 ~ 1.753)	0.015
≥10.95	0.927 (0.65 ~ 1.322)	0.675
Likelihood Ratio test		0.007

### 3.4. Subgroup analysis

**Fig 3:**
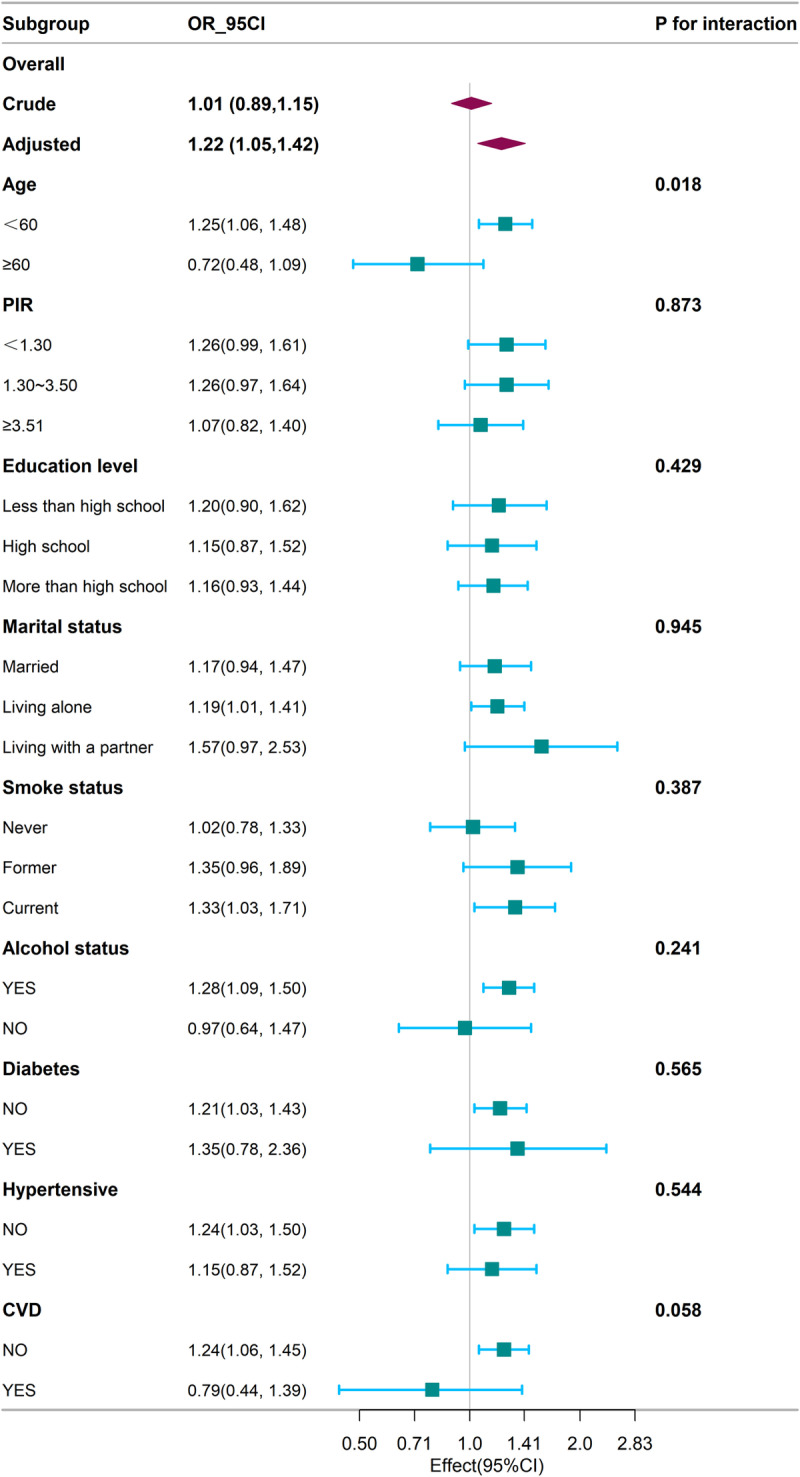
Subgroup analysis for the association between WWI and migraine.

Among male participants, we conducted weighted subgroup analyses and interaction tests stratified by age (<60 years, ≥ 60 years), poverty income ratio (PIR) (<1.30, 1.31–3.50, ≥ 3.51), educational attainment (less than high school, high school, more than high school), marital status (married, living alone, living with a partner), smoking status, alcohol consumption, diabetes, hypertension, and cardiovascular disease. These analyses examined whether the WWI-migraine association remained consistent across subgroups. According to the forest plot (Fig 3), a significant interaction was observed exclusively in the age-stratified subgroups (p for interaction = 0.018). A positive correlation between WWI and migraine was observed in participants under 60, living alone, current smokers, alcohol consumers, non-diabetics, non-hypertensive, and those without cardiovascular disease (p < 0.05).

### 3.5. ROC analysis

**Fig 4:**
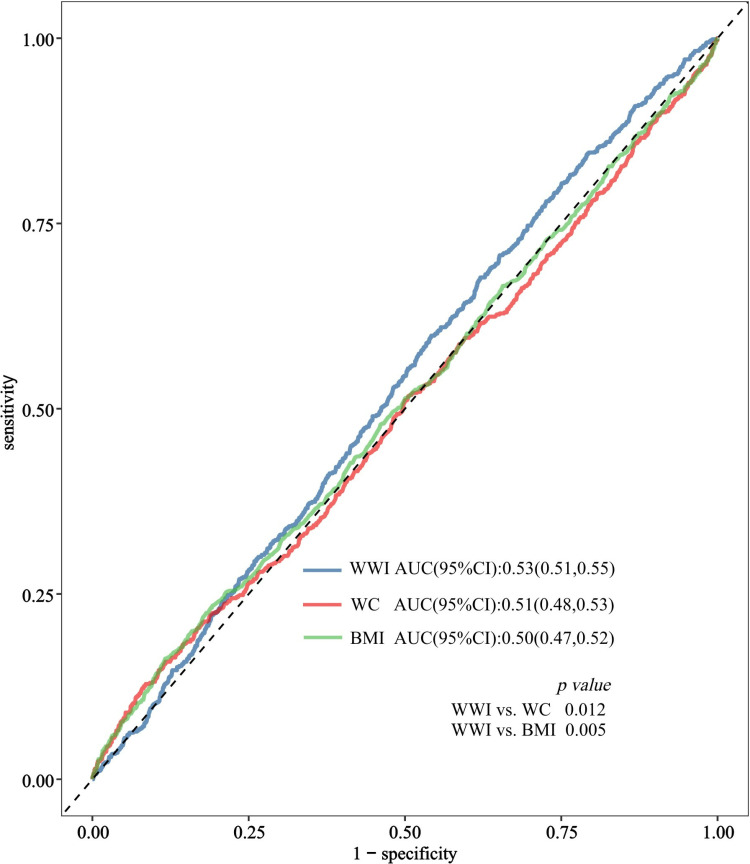
ROC curve analysis for predicting migraine.Delong’s test compares AUC.

ROC curves were generated, and the AUCs were determined to assess the predictive abilities of WWI, WC, and BMI for migraine (Fig 4). Our results showed that the AUC of WWI in predicting migraine was superior to that of both WC and BMI. The AUC differences between WWI and WC (p = 0.012) and between WWI and BMI (p = 0.005) were significant,suggesting that WWI serves as a more effective predictive marker for migraines than WC and BMI (Table 4).

**Table 4:** Performance of WWI and traditional obesity parameters in predicting the risk of migraine.

Variables	AUC	95%CI low	95%CI upp	Sensitivity	Specificity	Cutoff value	p for different in AUC
WWI	0.53	0.51	0.55	0.68	0.38	11.10	Reference
WC	0.51	0.48	0.53	0.16	0.88	83.55	0.012
BMI	0.50	0.47	0.52	0.13	0.91	21.66	0.005

## 4. Discussion

To our knowledge, this is one of the first studies to examine the association between WWI and migraine, providing new insights into how central obesity relates to migraine. In this cross-sectional analysis, we examined data from a representative group of 9,688 American adults (weighted population: 148,278,824) and found a significant difference between genders concerning the connection between WWI and migraine. Specifically, no significant association was found in female participants, whereas an inverted U-shaped non-linear positive correlation was evident in male participants. After adjusting for potential confounders, an increase of 1 unit in WWI was linked to a 22% rise in the risk of migraine among male participants，but no significant association was observed in females. Further analysis identified a threshold of 10.95 cm/√kg; notably, the risk of migraine increased with rising WWI up to this threshold, beyond which no further association was observed. Subgroup analysis revealed an interaction between age and the association of WWI with migraine in male participants.

Several studies investigating the relationship between obesity and migraine have yielded contradictory conclusions. Consistent with our findings in female participants, a large prospective study involving 19,162 middle-aged women revealed no substantial link between obesity and migraine over a follow-up duration of 12.9 years (HR 1.00, 95% CI: 0.83–1.19) [32]. Similarly, Mattson’s report involving 684 Swedish women aged 40–74 found no relationship between migraine and obesity [33]. All of the aforementioned studies are based on BMI as a measure of total body obesity (TBO). Research examining the association between central obesity and migraine has predominantly focused on WC. Peterlin et al. demonstrated a positive correlation between central obesity and the prevalence of migraine, but this correlation was restricted to individuals aged 55 years and younger. For those older than 55, the incidence of migraine was no longer linked to central obesity in males, while it decreased in females [34]. This finding aligns with the conclusions drawn from our male participants. In a health study involving 33,176 individuals from Norway, it indicated that individuals with central obesity—characterized by a WC exceeding 88 cm for women and 102 cm for men—faced a 29% heightened risk of migraine. Stratified analysis revealed that the risk of migraine escalated by 41% in males and 22% in females, with an increase of 89% for participants younger than 50 years and 26% for those aged 50 years and older [35]. It is noteworthy that these waist circumference (WC) cut-off values are derived from populations of European descent and may not universally apply to all racial and ethnic groups. Future research should incorporate racially adjusted anthropometric standards to enhance generalizability.Similarly, Rossoni de Oliveira et al. reported that migraine patients with elevated WC experienced a higher frequency of migraine attacks compared to those with normal WC [36].However, prior research has pointed out that BMI is not a dependable indicator for evaluating body fat distribution [37], and WC does not distinguish between subcutaneous and visceral fat. Notably, individuals with low BMI and WC levels can still exhibit visceral obesity [38]. WWI, a new and easily calculated metric of obesity, integrates the advantages of WC while mitigating the impact of BMI. It has been shown to have a positive relationship with fat content and an inverse relationship with muscle content, rendering it a more precise and thorough metric for evaluating abdominal obesity. Our study suggests that WWI has superior predictive capabilities for migraines in comparison to both BMI and WC.

The pathogenesis of migraine is not fully elucidated, but inflammation plays a significant role in its development [39]. Obesity, particularly the excessive accumulation of visceral adipose tissue (VAT), is strongly associated with increased chronic systemic inflammation [40]. WWI, as an indicator of central obesity, is closely related to VAT accumulation. Studies have shown that total VAT volume is significantly greater in men than in women [41]. There are significant sex differences in human fat distribution, which can powerfully predict disease risk [42,43]. In men, fat is primarily distributed in VAT around abdominal organs, resulting in an “apple-shaped” body, while women have more subcutaneous adipose tissue (SAT), leading to a “pear-shaped” distribution [44–46]. VAT has higher metabolic activity than SAT and can secrete more pro-inflammatory cytokines (such as IL-6 and TNF-α), thereby triggering systemic inflammation. [47–49]. Due to the higher proportion of SAT, lower pro-inflammatory state, and greater propensity to absorb circulating free fatty acids and triglycerides, women may have some protection against obesity-related diseases [50]. Furthermore, adipose tissue secretes various adipokines, such as leptin and adiponectin, which play important roles in inflammation and migraine. Leptin, as a pro-inflammatory factor, is typically higher in women, but increased VAT in men may lead to a more significant effect of leptin binding to its receptors, thereby promoting neuroinflammation [51]. Adiponectin has anti-inflammatory effects, and its levels are generally lower in men than in women and are negatively correlated with VAT [52], In men, elevated WHtR leads to increased VAT, decreased adiponectin levels, and reduced anti-inflammatory protection, which may increase the risk of migraine by enhancing inflammatory responses. Conversely, higher adiponectin levels in women may help maintain a lower inflammatory state, attenuating the association between WHtR and migraine. Calcitonin gene-related peptide (CGRP) is an important mediator of the trigeminovascular system, participating in migraine attacks by activating peripheral and central sensitization, neurogenic inflammation, and pain signal transmission [53]. Studies have shown that CGRP levels are significantly elevated in the plasma of obese individuals and are closely related to VAT accumulation [54]. In men, elevated WHtR may increase the risk of migraine by stimulating CGRP release through VAT-induced increases in leptin and decreases in adiponectin, thereby enhancing neuroinflammation and vascular reactivity.Sex hormones exhibit significant sexual dimorphism in regulating fat distribution and metabolic health. Studies have shown that estrogen exerts metabolic protective effects through multiple mechanisms: In terms of fat distribution, the metabolic protective effect of estrogen is not only reflected in reducing central/intra-abdominal fat accumulation but also in promoting the metabolic health of gluteofemoral subcutaneous fat depots. This optimization of regional fat distribution may be an important mechanism by which women have a relative metabolic advantage despite having a higher total fat mass [55–57]. From the perspective of adipose tissue function, estrogen (such as 17β-estradiol) improves white adipose tissue (WAT) function, reduces adipose tissue dysfunction in obesity and diabetes, promotes adiponectin secretion, and alleviates adipose tissue inflammation by inhibiting the release of pro-inflammatory cytokines (such as TNF-α and IL-6), thereby effectively reducing the chronic inflammatory response of visceral fat and the risk of related metabolic diseases [58]. This anti-inflammatory effect is closely related to the bidirectional effect of estrogen on immune regulation, that is, estrogen inhibits adipose tissue inflammation by upregulating anti-inflammatory pathways, while androgens exhibit immunosuppressive properties. This may explain the clinical observation that VAT in men is more likely to induce systemic inflammation and metabolic complications [57]. Therefore, the anti-inflammatory effect of estrogen may, to some extent, mask the effect of WHtR changes on migraine in women.

Our study possesses several strengths. It employed a sophisticated multistage probability sampling approach and benefited from a large number of samples, while controlling for various confounding factors. Moreover, we considered the dataset weights and used weighted analysis, which improved the reliability and representativeness of our results. Importantly, this investigation is the inaugural study to explore the possible connection between WWI and migraines in U.S. adults. In addition, we performed subgroup analyses to clarify the strength of the link between WWI and migraines in diverse populations.

Nonetheless, our research presents a few constraints.. First, due to the cross-sectional design of NHANES, we cannot establish a causal relationship between WWI and migraines. Second, there may be measurement errors associated with weight and WC. While the NHANES questionnaire effectively assesses disease status [59], the diagnosis of migraines primarily depends on self-reporting by participants, which inevitably introduces reporting bias and may compromise the accuracy of our conclusions. ‌Third we did not subtype migraines, which restricts our ability to analyze the manifestations of different types of migraines across various populations. Furthermore, Although many confounders were adjusted, due to limitations in NHANES data, we were unable to take into account all confounders (e.g., lifestyle, diet, hormones) and excluded the effects of unknown or non-measurable factors. Subsequent studies ought to emphasize implementing an extensive, longitudinal prospective investigation to to track adult men with different WWI levels, observe the relationship between changes in WWI and the occurrence of migraines over the long term, and assess whether WWI is an independent risk factor for migraines.

## 5. Conclusion

In conclusion, among U.S. adults, WWI is not linked to migraine risk in women; however, it exhibits an inverted U-shaped nonlinear positive correlation with migraine risk in men, with an inflection point at 10.95 cm/√kg. This association varies across different age groups. In comparison to BMI and WC, WWI shows enhanced predictive abilities for migraines, indicating that it could be a more effective anthropometric indicator.

Age,race,education level,marital status,PIR,smoking status,alcohol consumption, HB,CRP,TC, Diabetes, hypertension, kidney failure, CVD were adjusted. PIR,ratio of family income to poverty;HB,haemoglobin;CRP, C reactive protein;TC, Total cholesterol;CVD, Cardiovascular disease.

## References

[1] Lancet T GBD 2017: a fragile world. Lancet, 2018;392(10159): 1683. doi: 10.1016/S0140-6736(18)32858-7 30415747

[2] GBD 2015 Disease and Injury Incidence and Prevalence Collaborators. Global, regional, and national incidence, prevalence, and years lived with disability for 310 diseases and injuries, 1990-2015: a systematic analysis for the Global Burden of Disease Study 2015. Lancet. 2016;388(10053):1545–602. doi: 10.1016/S0140-6736(16)31678-6 27733282 PMC5055577

[3] Global, regional, and national burden of neurological disorders during 1990-2015: a systematic analysis for the Global Burden of Disease Study 2015. Lancet Neurol. 2017;16(11):877–97. 28931491 10.1016/S1474-4422(17)30299-5 PMC5641502

[4] Vos T, Flaxman AD, Naghavi M, Lozano R, Michaud C, Ezzati M, et al. Years lived with disability (YLDs) for 1160 sequelae of 289 diseases and injuries 1990-2010: a systematic analysis for the Global Burden of Disease Study 2010. Lancet. 2012;380(9859):2163–96. doi: 10.1016/S0140-6736(12)61729-2 23245607 PMC6350784

[5] Lipton RB, Stewart WF, Diamond S, Diamond ML, Reed M. Prevalence and burden of migraine in the United States: data from the American Migraine Study II. Headache. 2001;41(7):646–57. 11554952 10.1046/j.1526-4610.2001.041007646.x

[6] Burch R. Migraine and Tension-Type Headache: Diagnosis and Treatment. Med Clin North Am. 2019;103(2):215–33. doi: 10.1016/j.mcna.2018.10.003 30704678

[7] Jahromi SR, Martami F, Morad Soltani K, Togha M. Migraine and obesity: what is the real direction of their association?. Expert Rev Neurother. 2023;23(1):75–84. doi: 10.1080/14737175.2023.2173575 36714917

[8] Flegal KM, Kit BK, Orpana H, Graubard BI. Association of all-cause mortality with overweight and obesity using standard body mass index categories: a systematic review and meta-analysis. JAMA. 2013;309(1):71–82. doi: 10.1001/jama.2012.113905 23280227 PMC4855514

[9] Hainer V, Aldhoon-Hainerová I. Obesity paradox does exist. Diabetes Care. 2013;36 Suppl 2(Suppl 2):S276–81. doi: 10.2337/dcS13-2023 23882059 PMC3920805

[10] Uretsky S, Messerli FH, Bangalore S, Champion A, Cooper-Dehoff RM, Zhou Q, et al. Obesity paradox in patients with hypertension and coronary artery disease. Am J Med. 2007;120(10):863–70. doi: 10.1016/j.amjmed.2007.05.011 17904457

[11] Romero-Corral A, Somers VK, Sierra-Johnson J, Thomas RJ, Collazo-Clavell ML, Korinek J, et al. Accuracy of body mass index in diagnosing obesity in the adult general population. Int J Obes (Lond). 2008;32(6):959–66. doi: 10.1038/ijo.2008.11 18283284 PMC2877506

[12] Antonopoulos A, et al. From the BMI paradox to the obesity paradox: the obesity-mortality association in coronary heart disease. Obes Rev. 2016;17(10):989–1000. 27405510 10.1111/obr.12440

[13] Li C, Ford ES, McGuire LC, Mokdad AH. Increasing trends in waist circumference and abdominal obesity among US adults. Obesity (Silver Spring). 2007;15(1):216–24. doi: 10.1038/oby.2007.505 17228050

[14] Yusuf S, Hawken S, Ounpuu S, Bautista L, Franzosi MG, Commerford P, et al. Obesity and the risk of myocardial infarction in 27,000 participants from 52 countries: a case-control study. Lancet. 2005;366(9497):1640–9. doi: 10.1016/S0140-6736(05)67663-5 16271645

[15] Wang Y, Rimm EB, Stampfer MJ, Willett WC, Hu FB. Comparison of abdominal adiposity and overall obesity in predicting risk of type 2 diabetes among men. Am J Clin Nutr. 2005;81(3):555–63. doi: 10.1093/ajcn/81.3.555 15755822

[16] Park Y, Kim NH, Kwon TY, Kim SG. A novel adiposity index as an integrated predictor of cardiometabolic disease morbidity and mortality. Sci Rep. 2018;8(1):16753. doi: 10.1038/s41598-018-35073-4 30425288 PMC6233180

[17] Kim NH, Park Y, Kim NH, Kim SG. Weight-adjusted waist index reflects fat and muscle mass in the opposite direction in older adults. Age Ageing. 2021;50(3):780–6. doi: 10.1093/ageing/afaa208 33035293

[18] Qin Z, Chang K, Yang Q, Yu Q, Liao R, Su B. The association between weight-adjusted-waist index and increased urinary albumin excretion in adults: A population-based study. Front Nutr. 2022;9:941926. doi: 10.3389/fnut.2022.941926 36034904 PMC9412203

[19] Ye J, Hu Y, Chen X, Yin Z, Yuan X, Huang L, et al. Association between the weight-adjusted waist index and stroke: a cross-sectional study. BMC Public Health. 2023;23(1):1689. doi: 10.1186/s12889-023-16621-8 37658310 PMC10472709

[20] Lin Y. Relationship between weight-adjusted waist index and osteoporosis in the senile in the United States from the national health and nutrition examination survey, 2017-2020. J Clin Densitom. 2023;26(2):101361. 36922294 10.1016/j.jocd.2023.02.002

[21] Huang X-T, Lv X, Jiang H. The weight-adjusted-waist index and cognitive impairment among U.S. older adults: a population-based study. Front Endocrinol (Lausanne). 2023;14:1276212. doi: 10.3389/fendo.2023.1276212 38027119 PMC10663941

[22] Li M, Yu X, Zhang W, Yin J, Zhang L, Luo G, et al. The association between weight-adjusted-waist index and depression: Results from NHANES 2005-2018. J Affect Disord. 2024;347:299–305. doi: 10.1016/j.jad.2023.11.073 38000467

[23] Borrud L, Chiappa, M, Burt V, Gahche JJ, Zipf G, Dohrmann SM, et al. National health and nutrition examination survey: national youth fitness survey plan, operations, and analysis, 2012. 2014. 24709592

[24] Zipf G, Chiappa M, Porter KS, Ostchega Y, Lewis BG, Dostal J. National health and nutrition examination survey: plan and operations, 1999-2010. 2013. 25078429

[25] Xie R, Zhang Y. Association between 19 dietary fatty acids intake and rheumatoid arthritis: Results of a nationwide survey. Prostaglandins Leukot Essent Fatty Acids. 2023;188:102530. doi: 10.1016/j.plefa.2022.102530 36586398

[26] Zierfuss B, Höbaus C, Herz CT, Pesau G, Koppensteiner R, Schernthaner G-H. Predictive power of novel and established obesity indices for outcome in PAD during a five-year follow-up. Nutr Metab Cardiovasc Dis. 2020;30(7):1179–87. doi: 10.1016/j.numecd.2020.03.019 32451274

[27] Tao J, Zhang Y, Tan C, Tan W. Associations between weight-adjusted waist index and fractures: a population-based study. J Orthop Surg Res. 2023;18(1):290. doi: 10.1186/s13018-023-03776-8 37038167 PMC10088134

[28] Buse DC, Loder EW, Gorman JA, Stewart WF, Reed ML, Fanning KM, et al. Sex differences in the prevalence, symptoms, and associated features of migraine, probable migraine and other severe headache: results of the American Migraine Prevalence and Prevention (AMPP) Study. Headache. 2013;53(8):1278–99. doi: 10.1111/head.12150 23808666

[29] Tian S, Wu L, Zheng H, Zhong X, Liu M, Yu X, et al. Association between dietary folate intake and severe headache among adults in the USA: a cross-sectional survey. Br J Nutr. 2024;131(3):438–46. doi: 10.1017/S000711452300137X 37337781 PMC10784126

[30] Tian S, Yu X, Wu L, Zheng H, Zhong X, Xie Y, et al. Vitamin B6 and folate intake are associated with lower risk of severe headache or migraine in adults: An analysis based on NHANES 1999-2004. Nutr Res. 2024;121:51–60. doi: 10.1016/j.nutres.2023.11.008 38042023

[31] Curtin LR, Mohadjer LK, Dohrmann SM, Montaquila JM, Kruszan-Moran D, Mirel LB et al., The National Health and Nutrition Examination Survey: Sample Design, 1999-2006. Vital Health Stat. 2012; 2(155):1–39. 22788053

[32] Winter AC, Wang L, Buring JE, Sesso HD, Kurth T. Migraine, weight gain and the risk of becoming overweight and obese: a prospective cohort study. Cephalalgia. 2012;32(13):963–71. doi: 10.1177/0333102412455708 22875879 PMC3460066

[33] Mattsson P. Migraine headache and obesity in women aged 40-74 years: a population-based study. Cephalalgia. 2007;27(8):877–80. doi: 10.1111/j.1468-2982.2007.01360.x 17635528

[34] Peterlin BL, Rosso AL, Rapoport AM, Scher AI. Obesity and migraine: the effect of age, gender and adipose tissue distribution. Headache. 2010;50(1):52–62. doi: 10.1111/j.1526-4610.2009.01459.x 19496830 PMC3566428

[35] Kristoffersen ES, et al. Migraine, obesity and body fat distribution - a population-based study. J Headache Pain. 2020;21(1):97. 32762643 10.1186/s10194-020-01163-w PMC7409451

[36] Rossoni de Oliveira V, Camboim Rockett F, Castro K, da Silveira Perla A, Chaves MLF, Schweigert Perry ID. Body mass index, abdominal obesity, body fat and migraine features in women. Nutr Hosp. 2013;28(4):1115–20. doi: 10.3305/nh.2013.28.4.6504 23889629

[37] Elagizi A, Kachur S, Lavie CJ, Carbone S, Pandey A, Ortega FB, et al. An overview and update on obesity and the obesity paradox in cardiovascular diseases. Prog Cardiovasc Dis. 2018;61(2):142–50. 29981771 10.1016/j.pcad.2018.07.003

[38] Gulati S, Misra A. Abdominal obesity and type 2 diabetes in Asian Indians: dietary strategies including edible oils, cooking practices and sugar intake. Eur J Clin Nutr. 2017;71(7):850–7. 28612831 10.1038/ejcn.2017.92

[39] Liu J, Ren Q, Du B, Liu X, An Y, Zhang P, et al. Multi-omics approaches to deciphering complex pathological mechanisms of migraine: a systematic review. Front Pharmacol. 2025;15:1452614. doi: 10.3389/fphar.2024.1452614 39850553 PMC11754399

[40] Ahmed B, Farb MG, Gokce N. Cardiometabolic implications of adipose tissue aging. Obes Rev. 2024;25(11):e13806. 10.1111/obr.13806 39076025

[41] Demerath EW, Sun SS, Rogers N, Lee M, Reed D, Choh AC, et al. Anatomical patterning of visceral adipose tissue: race, sex, and age variation. Obesity (Silver Spring). 2007;15(12):2984–93. doi: 10.1038/oby.2007.356 18198307 PMC2883307

[42] Kautzky-Willer A, Harreiter J, Pacini G. Sex and Gender Differences in Risk, Pathophysiology and Complications of Type 2 Diabetes Mellitus. Endocr Rev. 2016;37(3):278–316. doi: 10.1210/er.2015-1137 27159875 PMC4890267

[43] Michaud A, Drolet R, Noël S, Paris G, Tchernof A. Visceral fat accumulation is an indicator of adipose tissue macrophage infiltration in women. Metabolism. 2012;61(5):689–98. doi: 10.1016/j.metabol.2011.10.004 22154325

[44] Fried SK, Lee M-J, Karastergiou K. Shaping fat distribution: New insights into the molecular determinants of depot- and sex-dependent adipose biology. Obesity (Silver Spring). 2015;23(7):1345–52. doi: 10.1002/oby.21133 26054752 PMC4687449

[45] Schwartz RS, Shuman WP, Larson V, Cain KC, Fellingham GW, Beard JC, et al. The effect of intensive endurance exercise training on body fat distribution in young and older men. Metabolism. 1991;40(5):545–51. doi: 10.1016/0026-0495(91)90239-s 2023542

[46] Link JC, Hasin-Brumshtein Y, Cantor RM, Chen X, Arnold AP, Lusis AJ, et al. Diet, gonadal sex, and sex chromosome complement influence white adipose tissue miRNA expression. BMC Genomics. 2017;18(1):89. doi: 10.1186/s12864-017-3484-1 28095800 PMC5240420

[47] Geer EB, Shen W. Gender differences in insulin resistance, body composition, and energy balance. Gend Med. 2009;6 Suppl 1(Suppl 1):60–75. doi: 10.1016/j.genm.2009.02.002 19318219 PMC2908522

[48] Stevens J, Katz EG, Huxley RR. Associations between gender, age and waist circumference. Eur J Clin Nutr. 2010;64(1):6–15. doi: 10.1038/ejcn.2009.101 19738633 PMC5909719

[49] Smith SR, Lovejoy JC, Greenway F, Ryan D, deJonge L, de la Bretonne J, et al. Contributions of total body fat, abdominal subcutaneous adipose tissue compartments, and visceral adipose tissue to the metabolic complications of obesity. Metabolism. 2001;50(4):425–35. doi: 10.1053/meta.2001.21693 11288037

[50] Ibrahim M. Subcutaneous and visceral adipose tissue: structural and functional differences. Obes Rev. 2010;11(1):11–8. 19656312 10.1111/j.1467-789X.2009.00623.x

[51] Peterlin BL, Sacco S, Bernecker C, Scher AI. Adipokines and Migraine: A Systematic Review. Headache. 2016;56(4):622–44. 27012149 10.1111/head.12788 PMC4836978

[52] Chávez-Guevara IA, Amaro-Gahete FJ, Osuna-Prieto FJ, Labayen I, Aguilera CM, Ruiz JR. The role of sex in the relationship between fasting adipokines levels, maximal fat oxidation during exercise, and insulin resistance in young adults with excess adiposity. Biochem Pharmacol. 2023;216:115757. doi: 10.1016/j.bcp.2023.115757 37598975

[53] Zhu Q, Yang J, Shi L, Zhang J, Zhang P, Li J, et al. Exploring the role of ubiquitination modifications in migraine headaches. Front Immunol. 2025;16:1534389. doi: 10.3389/fimmu.2025.1534389 39958329 PMC11825825

[54] Timper K, Grisouard J, Radimerski T, Dembinski K, Peterli R, Häring A, et al. Glucose-dependent insulinotropic polypeptide (GIP) induces calcitonin gene-related peptide (CGRP)-I and procalcitonin (Pro-CT) production in human adipocytes. J Clin Endocrinol Metab. 2011;96(2):E297-303. doi: 10.1210/jc.2010-1324 21106708

[55] Bouchard C, Després JP, Mauriège P. Genetic and nongenetic determinants of regional fat distribution. Endocr Rev. 1993;14(1):72–93. doi: 10.1210/edrv-14-1-72 8491156

[56] Zhu B-T, Liao Q-Q, Tian H-Y, Yu D-J, Xie T, Sun X-L, et al. Estrogen: the forgotten player in metaflammation. Front Pharmacol. 2024;15:1478819. doi: 10.3389/fphar.2024.1478819 39575382 PMC11578702

[57] Yalcinkaya A, Yalcinkaya R, Sardh F, Landegren N. Immune dynamics throughout life in relation to sex hormones and perspectives gained from gender-affirming hormone therapy. Front Immunol. 2024;15:1501364. 39885993 10.3389/fimmu.2024.1501364 PMC11779622

[58] Martínez-Cignoni MR, González-Vicens A, Morán-Costoya A, Amengual-Cladera E, Gianotti M, Valle A, et al. Diabesity alters the protective effects of estrogens on endothelial function through adipose tissue secretome. Free Radic Biol Med. 2024;224:574–87. doi: 10.1016/j.freeradbiomed.2024.09.001 39241985

[59] Lopez-Jimenez F, Batsis JA, Roger VL, Brekke L, Ting HH, Somers VK. Trends in 10-year predicted risk of cardiovascular disease in the United States, 1976 to 2004. Circ Cardiovasc Qual Outcomes. 2009;2(5):443–50. 20031875 10.1161/CIRCOUTCOMES.108.847202 PMC2779550

